# Supplementary material for: Women with Cervical High-Risk Human Papillomavirus: Be Aware of Your Anus! The ANGY Cross-Sectional Clinical Study
Source: Cancers (Basel). 2022 Oct 18;14(20):5096. doi: 10.3390/cancers14205096 (PMC9600245; doi:10.3390/cancers14205096)
Supplement: Supplementary file 1 [file cancers-14-05096-s001.zip › cancers-1899040-supplementary.pdf]

Supplementary File S1

Questionnaire for the participants of the ANGY study

1. Have you ever been pregnant? (Y/N) If yes, how many times? (number)

1. Have you had children? (Y/N) If yes, how many children do you have? (number)

2. Schooling and studies: Secondary school, Apprenticeship, Gymnasium/high school, HES, University

3. Are you taking medication for cancer or because of an organ transplant? (Y/N)

4. Are you a carrier of the AIDS virus (HIV)? (Y/N/dont' know)

5. Do you smoke cigarettes? (Y/N) If yes: number of cigarettes/day and for how many years (number).

6. How many sexual partners have you had? 0, 1, 2-5, 5-10, >10

7. At what age did you have your first sexual intercourse? (year)

8. Do you always have sex with a condom? (Y/N)

9. Have you ever had anal sex (sodomy)? (Y/N) If yes, in the past 6 months? (Y/N)

10. Have you had any anal or anal canal disease? (Y/N) If yes: Anal fistula, Anal fissure, Hemorrhoids, Other.

11. Have you been vaccinated against the HPV virus? (Y/N) If yes, which vaccine? How many injections?

12. Are you taking birth control (Y/N) If yes, which one?

13. Have you ever had an abnormal annual Pap smear? (Y/N)

14. Have you ever heard of anal cancer? (Y/N)

Supplementary Table S1. Multivariate analysis of factors associated with cervical HR-HPV

Variable	ORa	IC 95%	p
Age (per year)	0.96	0.92 – 0.99	0.02
Parity (per child)	0.92	0.67 – 1.27	0.62
Age of first sexual intercourse (per year)	1.02	0.88 – 1.20	0.77
Anal intercourse			
No	1		
Yes	0.83	0.35 – 1.98	0.68
Number of sexual partners			
0	1		
1	0.13	0.01 – 2.02	0.15
2-4	0.30	0.02 – 3.94	0.36
5-10	0.73	0.06 – 9.48	0.81
> 10	0.99	0.08 – 12.69	0.99
Smoking			
No	1		
Yes	2.53	1.08 – 5.94	0.03
Anal LR HPV			
No	1		
Yes	1.14	0.49 – 2.63	0.77
Anal HR HPV			
No	1		
Yes	2.47	1.01 – 6.06	**0.048**
Anal cytology			
Normal	1		
LSIL	1.05	0.16 – 6.80	0.96
HSIL	2.29	0.23 – 22.53	0.48
ASCUS	1.40	0.25 – 7.94	0.70
ASC-H	1.94	0.04 – 98.25	0.74
Cervical LR HPV			
No	1		
Yes	3.23	1.30 – 8.01	0.01
Cervical cytology			
Normal	1		
LSIL	3.58	0.80 – 16.10	0.1
HSIL	3.21	0.60 – 17.30	0.17

ASCUS	1.45	0.43 – 4.82	0.55
ASC-H	3.51	0.48 – 25.63	0.22

HR-HPV: High-Risk Human Papillomavirus. LR-HPV: Low Risk Human Papillomavirus. PCR: Polymerase Chain Reaction. LSIL: Low grade squamous intraepithelial lesion. HSIL: High grade squamous intraepithelial lesion. ASCUS: Atypical squamous cells of undetermined significance. ASC-H: Atypical squamous cells-cannot exclude high-grade squamous intraepithelial lesion. Significant p-value < 0.05 are in bold characters
